# A Randomized, Placebo-Controlled, Active-Reference, Double-Blind, Flexible-Dose Study of the Efficacy of Vortioxetine on Cognitive Function in Major Depressive Disorder

**DOI:** 10.1038/npp.2015.52

**Published:** 2015-04-01

**Authors:** Atul R Mahableshwarkar, John Zajecka, William Jacobson, Yinzhong Chen, Richard SE Keefe

**Affiliations:** 1 Takeda Development Center Americas, Deerfield, IL, USA; 2 Department of Psychiatry, Rush University Medical Center, Chicago, IL, USA; 3 Department of Psychiatry and Behavioral Sciences, Duke University Medical Center, Durham, NC, USA

## Abstract

This multicenter, randomized, double-blind, placebo-controlled, active-referenced (duloxetine 60 mg), parallel-group study evaluated the short-term efficacy and safety of vortioxetine (10–20 mg) on cognitive function in adults (aged 18–65 years) diagnosed with major depressive disorder (MDD) who self-reported cognitive dysfunction. Efficacy was evaluated using ANCOVA for the change from baseline to week 8 in the digit symbol substitution test (DSST)–number of correct symbols as the prespecified primary end point. The patient-reported perceived deficits questionnaire (PDQ) and physician-assessed clinical global impression (CGI) were analyzed in a prespecified hierarchical testing sequence as key secondary end points. Additional predefined end points included the objective performance-based University of San Diego performance-based skills assessment (UPSA) (ANCOVA) to measure functionality, MADRS (MMRM) to assess efficacy in depression, and a prespecified multiple regression analysis (path analysis) to calculate direct *vs* indirect effects of vortioxetine on cognitive function. Safety and tolerability were assessed at all visits. Vortioxetine was statistically superior to placebo on the DSST (*P*<0.05), PDQ (*P*<0.01), CGI-I (*P*<0.001), MADRS (*P*<0.05), and UPSA (*P*<0.001). Path analysis indicated that vortioxetine's cognitive benefit was primarily a direct treatment effect rather than due to alleviation of depressive symptoms. Duloxetine was not significantly different from placebo on the DSST or UPSA, but was superior to placebo on the PDQ, CGI-I, and MADRS. Common adverse events (incidence ⩾5%) for vortioxetine were nausea, headache, and diarrhea. In this study of MDD adults who self-reported cognitive dysfunction, vortioxetine significantly improved cognitive function, depression, and functionality and was generally well tolerated.

## Introduction

Major depressive disorder (MDD) is the most common psychiatric disorder, with a lifetime prevalence of 16.2% and a 12-month prevalence of 6.6% in developed countries (Trivedi *et al*, 2007). It represents one of the most serious challenges faced by health-care providers and is a leading cause of disability (World Health Organization (WHO), 2009). MDD is characterized by the presence of one or more major depressive episodes (MDEs), presenting with depressed mood, loss of interest or pleasure, disturbed sleep or appetite, low energy, and feelings of guilt or low self-worth (Uher *et al*, 2014).

Mood disorders are also associated with impairments in cognitive functioning. A growing body of evidence from neuropsychological studies suggests that many patients suffering from MDD present with some form of dysfunction in certain cognitive domains, such as executive function, working memory, visuospatial short-term memory, immediate and delayed free recall, psychomotor speed, and verbal learning (Bora *et al*, 2013; Gualtieri and Morgan, 2008; Hill *et al*, 2004; Lee *et al*, 2012; Mahurin *et al*, 2006; Murrough *et al*, 2011; Robinson *et al*, 2006; Rock *et al*, 2014; Smith *et al*, 2006; Stordal *et al*, 2004; Zakzanis *et al*, 1998). In one study (Burt *et al*, 1995), patients with depression performed approximately one-half of SD worse than healthy subjects on verbal learning and memory tests. Other studies, however, found nonsignificant or only minimal differences in neuropsychological tests between patients with MDD and healthy controls (Castaneda *et al*, 2008; Grant *et al*, 2001; Miller *et al*, 1991).

Vortioxetine is an approved antidepressant for the treatment of adult patients with MDD and is thought to work through a combination of two pharmacological modes of action: serotonin (5-HT) reuptake inhibition and direct activity at 5-HT receptors. In recombinant cell lines, vortioxetine shows 5-HT_3_, 5-HT_7_, and 5-HT_1D_ receptor antagonism, 5-HT_1A_ receptor agonism, and 5-HT_1B_ receptor partial agonism, and is an inhibitor of the 5-HT transporter (Westrich *et al*, 2012). The *in vivo* nonclinical studies in rodents have demonstrated that vortioxetine modulates serotonergic, noradrenergic, dopaminergic, cholinergic, histaminergic, and glutamatergic neurotransmission, affecting the levels of neurotransmitters involved in cognitive processes (Bang-Andersen *et al*, 2011; Mork *et al*, 2012, 2013; Pehrson *et al*, 2013; Pehrson and Sanchez, 2014; Wallace *et al*, 2014). Evidence from recent preclinical cognitive behavioral models suggests vortioxetine has a beneficial effect on cognition (Pehrson *et al*, 2014; Sanchez *et al*, 2015).

Clinical evidence from a double-blind, placebo-controlled, duloxetine-referenced study in elderly (≥65 years old) MDD patients demonstrated superiority for vortioxetine compared with placebo in predefined objective neuropsychological tests of speed of processing, verbal learning, and memory (Katona *et al*, 2012). Results from a recently published double-blind, placebo-controlled study further demonstrated the clinical benefits of vortioxetine on cognitive functioning in adults with MDD (McIntyre *et al*, 2014). In addition, exploratory and *post hoc* analyses provided further support of the hypothesis that vortioxetine improves cognitive functioning in subjects with MDD (Keefe *et al*, 2013b).

The primary objective of this multicenter, double-blind, parallel-group, placebo-controlled, active-referenced study in subjects with acute recurrent MDD was to compare the effect of vortioxetine with placebo on cognitive functioning, including specific measures of attention, executive functioning, and psychomotor speed. A secondary objective was to assess the efficacy of vortioxetine *vs* placebo on depressive symptoms and functional capacity.

## Materials and Methods

Subjects with MDD who subjectively reported cognitive dysfunction were randomly assigned to receive 8 weeks of double-blind treatment comparing flexible doses of vortioxetine (10 or 20 mg q.d.) or placebo. Duloxetine 60 mg q.d. was included as the active reference arm to demonstrate assay sensitivity to traditional antidepressant outcomes. A 1-week, double-blind taper-down period was implemented following acute treatment phase to address potential concerns regarding discontinuation symptoms with duloxetine treatment (see Supplementary Appendix A). The study was conducted between April 2012 and February 2014, enrolling a total of 602 subjects at 80 psychiatric inpatient and outpatient sites in the United States and Europe using doses in line with current approved prescribing information.

All subjects who entered the trial reviewed and signed an informed consent document explaining study procedures and potential risks before study entry. The study protocol and all related forms and amendments were approved by the independent ethics committee of each study center. The study was conducted in accordance with the International Conference on Harmonization Good Clinical Practices guidelines and with the ethical principles of the Declaration of Helsinki. This study is registered at ClinicalTrials.gov with registration number NCT01564862.

### Participants

Subjects were 18 to 65 years of age and met the Diagnostic and Statistical Manual of Mental Disorders, Fourth Edition, Text Revision (DSM-IV-TR) diagnosis of MDD as the only axis-I diagnosis. Subjects were required to have a diagnosis of acute MDE in the context of recurrent MDD. Evidence of a current MDE was confirmed using the Mini International Neuropsychiatric Interview (MINI) and through assessment of past medical records. Subjects had to have moderate to severe depression, with a Montgomery–Åsberg Depression Rating Scale (MADRS) total score of ≥26 at screening and baseline, and a duration of at least 3 months for the current MDE. Subjects with a history of lack of response to duloxetine were excluded. In addition, subjects were required to have self-reported subjective cognitive dysfunction (such as difficulty concentrating, slow thinking, and difficulty in learning new things or remembering things) during the intake interview. All subjects were evaluated at baseline using the Digit Symbol Substitution Test–number of correct entries (DSST performance), with a required baseline score of <70 to avoid any ceiling effect. A full listing of inclusion and exclusion criteria is available at www.clinicaltrials.gov/ct2/show/NCT01564862.

### Study Medication

At baseline (day 0), subjects who continued to meet all study inclusion and none of the exclusion criteria were randomly assigned via an interactive voice response system (in a 1 : 1 : 1 ratio) to one of the three treatment arms: vortioxetine, duloxetine, or placebo. Study medication was administered in the morning with or without food.

Subjects assigned to vortioxetine received 10 mg/day on days 1–7 of the double-blind treatment period, with the option to increase to vortioxetine 20 mg/day at the end of week 1 based on investigator judgment. For the remaining 7 weeks, the dose of vortioxetine was flexible at 10 or 20 mg/day based on investigator judgment. Subjects assigned to vortioxetine received placebo during the taper-down period. Subjects assigned to the placebo arm received placebo for the 8-week double-blind period as well as the taper-down period. Subjects assigned to the active reference arm received duloxetine 60 mg/day for the duration of the 8-week double-blind treatment period and duloxetine 30 mg/day for the 1-week taper-down period. The duloxetine dosage of 60 mg/day was consistent with the duloxetine package insert (http://pi.lilly.com/us/cymbalta-pi.pdf) that states that efficacy in MDD has been demonstrated in a dosage range of 40–60 mg/day, with higher doses not demonstrated to be more efficacious and associated with dose-dependent adverse events. Taper-down study medication was also offered to all subjects who withdrew prematurely (see Supplementary Appendix A).

### Assessments

The screening evaluation consisted of the MINI, medical and psychiatric histories, physical examination, measurement of vital signs, electrocardiogram (ECG), evaluation of suicidality using the Columbia Suicide Severity Rating Scale (C-SSRS), and clinical laboratory tests.

Efficacy was assessed using a battery of objective tests of cognitive function representing multiple domains: DSST performance (integrated cognitive functioning, including executive function, processing speed, attention, spatial perception, and visual scanning), Trail Making Test A (speed of processing), Trail Making Test B (executive functioning), Stroop Test (executive functioning), Groton Maze Learning Test (visual learning and memory), Detection Task (motor speed), Identification Task (attention), and One-Back Task (attention, working memory). Also used were subjective patient-reported assessments of cognitive function: perceived deficits questionnaire (PDQ) and cognitive and physical functioning questionnaire (CPFQ); assessments of depressive symptoms: MADRS (depressive symptoms) and Clinical Global Impressions (CGI, global clinical status); and objective and subjective assessments of overall functionality: University of California, San Diego performance-based skills assessment (UPSA, performance measures of functional capacity), and working limitation questionnaire (WLQ, patient-reported workplace productivity). The testing hierarchy of primary, predefined key secondary and additional predefined end points is described in Supplementary Appendix B. Safety and tolerability evaluations included vital signs and weight, physical examination, clinical safety laboratory tests, ECGs, and reported adverse events (AEs). Suicidality was evaluated by the C-SSRS at each study visit through the end of the acute treatment phase (week 8) or subject withdrawal.

### Statistical Analysis

A statistical testing strategy was defined *a priori* to control for multiplicity and comprised the primary efficacy analysis as well as the key secondary efficacy analyses. To control for type I error, the following sequence of hierarchically ordered primary and key secondary end points was used at a significance level of 0.05:

- Change from baseline to week 8 between vortioxetine and placebo in DSST performance score
- Change from baseline to week 8 between vortioxetine and placebo in PDQ attention/concentration and planning/organization subscore
- Clinical Global Impressions - Improvement (CGI-I) score at week 8

The change from baseline in DSST performance score after 8 weeks of treatment was analyzed using analysis of covariance (ANCOVA) in observed cases (OC), with treatment and pooled center as fixed factors, and baseline DSST performance as covariate. The change from baseline in the CGI-I (using the Clinical Global Impressions-Severity [CGI-S] score at baseline for reference), the CPFQ, and the PDQ attention/concentration and planning/organization subscores were analyzed using the mixed model for repeated measures (MMRM) using all available data. Change from baseline in the four WLQ scale scores and percentage of productivity loss score after 8 weeks of treatment was analyzed using an ANCOVA model similar to the one described for the primary variable. The CPFQ was also analyzed in the subgroup of subjects with a baseline CPFQ score >25, representing a self-perception of greater than minimal cognitive or physical dysfunction. The UPSA-VIM score and UPSA-Brief score were derived separately, and then combined as a unique score and analyzed together. Change from baseline in the UPSA composite score after 8 weeks of treatment was analyzed using ANCOVA (OC).

A power calculation was obtained via computer simulations on the primary outcome of difference in the change from baseline in DSST performance score between vortioxetine and placebo. Assuming SD of 10.8 for the change from baseline in the DSST performance score at week 8 and a 15% dropout rate, it was calculated that 600 subjects (200 per treatment group) were required to achieve ≥80% power to detect a difference of 3.3 in the change from baseline in DSST performance score between vortioxetine and placebo by a 2-sample *t*-test with a 2-sided significance level of *P*<0.05. The study was not designed to detect a statistical difference between vortioxetine and duloxetine on the primary or secondary outcome measures.

The primary analysis was performed utilizing ANCOVA and using the full analysis set (FAS), with treatment and center as fixed factors and baseline score as a covariate. The FAS included all subjects who were randomized, received at least one dose of study drug, and had at least one valid postbaseline value for assessment of the primary end point. Secondary efficacy analyses were conducted at all time points (where rated) using an ANCOVA model similar to that described for the primary end point. Cohen's *d* was used to estimate effect sizes and was calculated as the mean difference from placebo divided by the SD of the mean difference. A predefined path analysis was performed on the FAS (ANCOVA, OC) to address the potential issue of pseudospecificity and assess the extent to which improvement in cognitive functioning, as measured by DSST performance, was a direct treatment effect *vs* an indirect effect mediated through a general improvement of depressive symptoms, as measured by the change in MADRS. The effect estimates in the path analysis were determined using two ANCOVA models. In the first ANCOVA model, the direct effect of vortioxetine on cognitive deficits was determined based on estimates from a model adjusting for the correlation between changes in MADRS total score and the cognitive function assessment tool scores. The indirect effect, passing through change in depressive symptoms, was calculated by multiplying the correlation estimate from the first ANCOVA model with the estimates from the second ANCOVA model that estimated the effect of vortioxetine on depressive symptoms using the MADRS total score. The direct and indirect effects are presented as percentages of the total effect (direct effect+indirect effect). The C-SSRS was summarized at all time points for each treatment group using descriptive techniques. All *P*-values, least-squares (LS) treatment means, differences between the LS treatment means, and 95% confidence intervals (CIs) for the treatment differences were displayed using two-sided *t*-tests at the 5% level of significance comparing vortioxetine with placebo. The duloxetine treatment group was compared with placebo using the same analysis model, without the multiple comparison adjustment. No statistical assessment was performed on any safety or tolerability parameter.

## Results

**Figure 1:**
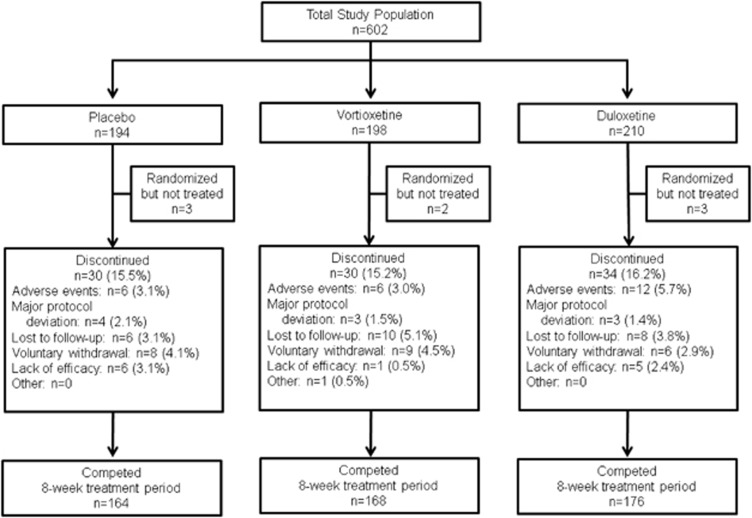
Study flow of a randomized, double-blind, placebo-controlled, and duloxetine-referenced study of vortioxetine.

**Figure 2:**
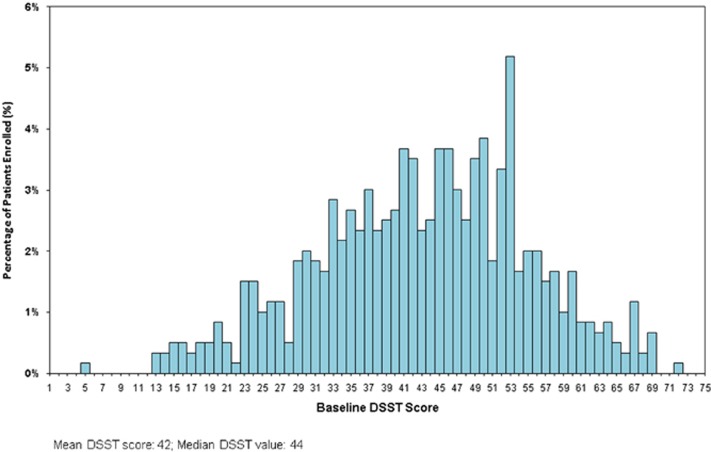
Distribution of DSST number of correct symbols score at baseline.

A total of 602 MDD subjects with self-reported cognitive dysfunction were randomized at 80 psychiatric inpatient and outpatient centers (Bulgaria, 10 sites, *n*=127; Finland, 2 sites, *n*=9; Germany, 7 sites, *n*=90; Poland, 4 sites, *n*=40; Russia, 3 sites, *n*=11; Ukraine, 3 sites, *n*=19; and the United States, 51 sites, *n*=306) to receive double-blind treatment (Figure 1). Baseline demographics and clinical characteristics, including disease severity, duration of current episode, number of previous MDEs, and overall severity, were similar across the three treatment arms (Table 1). The mean baseline DSST performance score of the total study population was 43.1 (median value, 44) with a limited number of high performers with baseline DSST performance scores approaching the upper limit of 70 minimizing ceiling effect (Figure 2). The proportion of subjects in the vortioxetine group who completed the 8-week treatment period (84.8%) was similar to the placebo group (84.5%) and the duloxetine group (83.8%) (Figure 1). The mean dose of vortioxetine during the study was 16.0 mg, with the dose of duloxetine fixed at 60 mg for the entire duration of the trial.

### Primary Analysis

**Figure 3:**
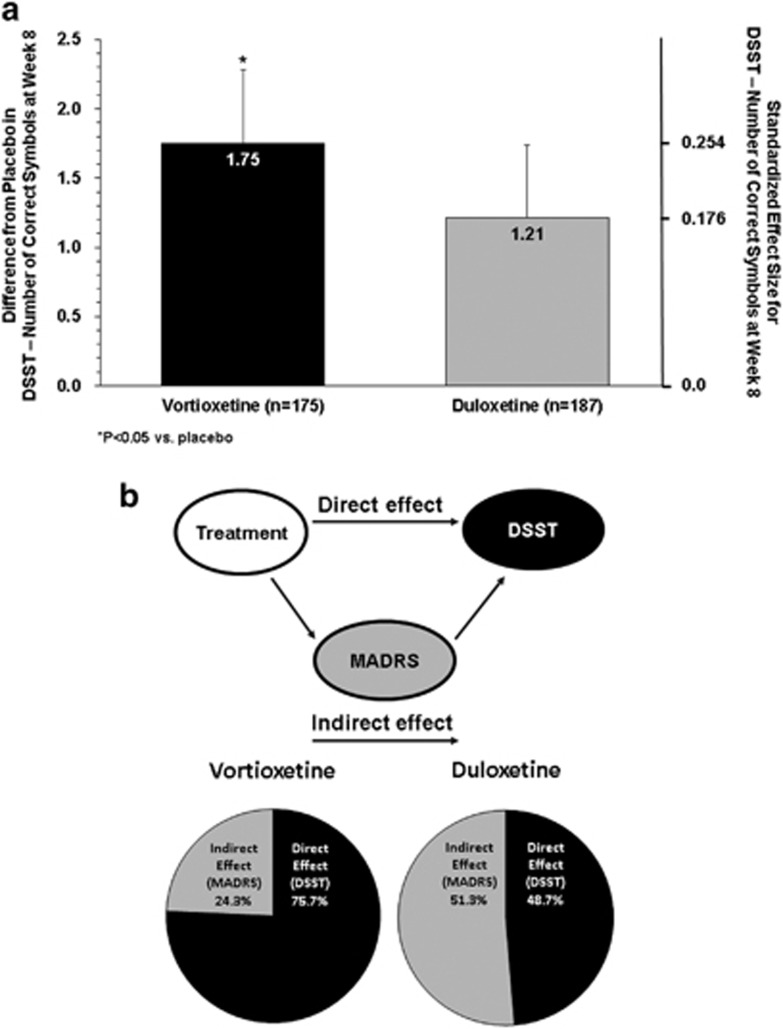
(a) Difference from placebo and standardized effect size *vs* placebo in DSST number of correct symbols at week 8 (ANCOVA, OC, LS means). (b) Path analysis of direct and indirect effects on cognitive function.

Based on the ANCOVA analysis, the change from baseline (mean±SE) to week 8 in DSST performance score was 4.60±0.53 for vortioxetine, 4.06±0.51 for duloxetine, and 2.85±0.54 for placebo. The difference from placebo was significant for vortioxetine (Δ +1.75, 95% CI: 0.28, 3.21; *P*=0.019; ANCOVA, OC), with a standardized effect size of 0.254 (Table 2 and Figure 3). The difference from placebo was not significant for the duloxetine group (Δ +1.21, 95% CI: −0.23, 2.65; *P*=0.099), with a standardized effect size of 0.176.

### Key Secondary Outcomes

**Figure 4:**
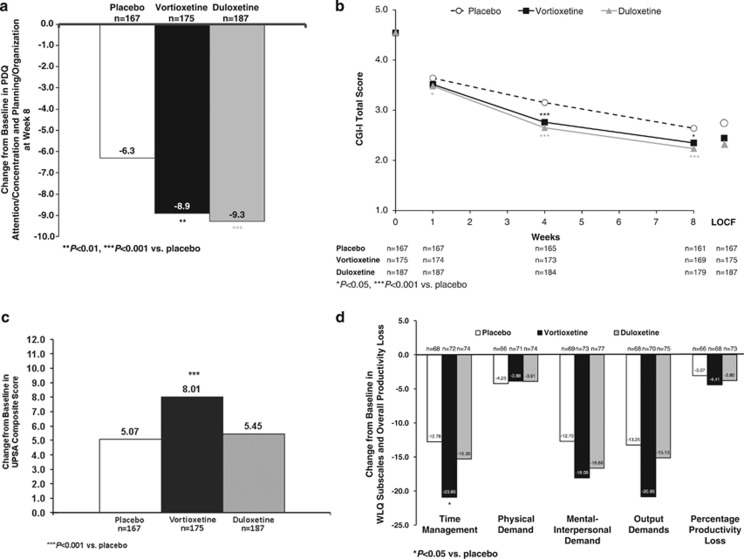
(a) Change from baseline in PDQ attention/concentration and planning/organization score (MMRM, FAS, LS means) at week 8. (b) CGI-I score by assessment visit (MMRM, FAS, LS means). (c) Change from baseline in UPSA total score at week 8 (ANCOVA, OC, LS means). (d) Change from baseline in WLQ subscores and percentage productivity loss at week 8 (ANCOVA, OC, LS means).

Both vortioxetine and duloxetine produced statistically significant improvement in the PDQ attention/concentration and planning/organization subscore, a subjective patient-reported outcome measure of cognitive function, as measured by the difference from placebo at week 8 (vortioxetine, Δ −2.6, 95% CI: −4.1, −1.0; *P*=0.001; duloxetine, Δ −3.0, 95% CI: −4.5, −1.5; *P*<0.001; MMRM, FAS) (Table 2 and Figure 4a). Vortioxetine produced a statistically significant improvement in disease severity compared with placebo in reducing the CGI-I score at week 8 (Δ −0.29, 95% CI: −0.53, −0.05; *P*<0.05) (Figure 4b). Treatment with duloxetine also demonstrated a statistically significant improvement on the CGI-I compared with placebo at week 8 (Δ −0.40, 95% CI: −0.64, −0.17; *P*<0.001; MMRM, FAS).

### Additional Cognitive Function Assessments

Trail Making Test B Total Time (Δ −9.67, 95% CI: −15.38, −3.96; *P*<0.001; ANCOVA, OC) was significantly improved with vortioxetine compared with placebo. None of the other secondary end points of cognitive function improved significantly with vortioxetine. No significant benefit was found with duloxetine compared with placebo on any of the secondary measures of cognitive function.

An additional analysis of the comparative effects of vortioxetine and duloxetine was conducted on the change from baseline on DSST total number of correct symbols at week 8. The effect of vortioxetine was not significantly different from duloxetine (Δ +0.54, 95% CI: −0.89, 1.96; *P*=0.46; ANCOVA, OC).

### Additional Functionality Assessments

Vortioxetine demonstrated a significant improvement in UPSA composite score compared with placebo at week 8 (*n*=175, Δ +2.94, 95% CI: 1.35, 4.52; *P*<0.001; ANCOVA, OC) (Table 2 and Figure 4c). Significant improvement was also noted on the UPSA–Brief (*n*=97, Δ +4.02, 95% CI: 1.63, 6.41; *P*=0.001) but not the UPSA–Validation of Intermediate Measures (VIM) (*n*=78, Δ +1.75, 95% CI: −0.20, 3.71; *P*=0.078). Treatment with duloxetine did not yield a significant change in UPSA composite score (*n*=187, Δ +0.38, 95% CI: −1.19, 1.94; *P*=0.637), UPSA–Brief (*n*=93, Δ −0.35, 95% CI: −2.76, 2.06; *P*=0.775), or UPSA–VIM (*n*=94, Δ +1.17, 95% CI: −0.70, 3.04; *P*=0.219) compared with placebo at week 8. An additional analysis of the comparative effects of vortioxetine and duloxetine was conducted on the change from baseline on the UPSA composite score at week 8. Vortioxetine demonstrated a significantly greater improvement on the UPSA composite score compared with duloxetine (Δ +2.56, 95% CI: 1.03, 4.10; *P*=0.001; ANCOVA, OC).

Vortioxetine did not demonstrate a significant difference from placebo on the CPFQ total score at week 8 (Δ −1.2, *P*=0.086; MMRM, FAS) for all patients (*N*=175) but did demonstrate a significant change in those subjects with self-perception of greater than minimal dysfunction (CPFQ >25) (*n*=139, Δ −1.7, *P*=0.041) (Table 2). Treatment with duloxetine demonstrated a significant improvement over placebo on CPFQ total score overall (*n*=187, Δ −1.7, *P*=0.012) as well as in subjects with baseline CPFQ >25 (*n*=147, Δ −1.8, *P*=0.024).

Neither vortioxetine nor duloxetine demonstrated an improvement in workplace productivity for the subset of working subjects compared with placebo (*n*=73/175 (41.7%); *n*=77/187 (41.2%); *n*=69/167 (41.3%), respectively) according to the measure of percent of productivity loss score on the WLQ at week 8. Vortioxetine was significantly superior to placebo in decreasing the time management score (Δ −8.13, *P*=0.045) (Table 2 and Figure 4d).

### Depression Outcome

The study was validated because both vortioxetine and duloxetine demonstrated a statistically significant change from baseline in mood symptoms compared with placebo at the end of week 8, as measured by change in MADRS (vortioxetine, Δ −2.3, 95% CI: −4.3, −0.4; *P*<0.05; duloxetine, Δ −3.3, 95% CI: −5.2, −1.4; *P*<0.001; MMRM, FAS) (Table 2 and Supplementary Appendix C).

### Path Analysis

Path analysis demonstrated that 75.7% of the effect of vortioxetine on cognitive functioning (DSST performance) could be directly attributed to an independent treatment effect and was not mediated by improvements in mood or depressive symptoms (MADRS). The direct and indirect effects of duloxetine on cognitive function were 48.7% and 51.3%, respectively (Figure 3b).

### Safety

A comparable proportion of subjects in the vortioxetine group (59.7%) and in the duloxetine group (57.5%) reported at least one treatment-emergent adverse event (TEAE); the proportion was somewhat lower in the placebo group (44.5%). The majority of TEAEs in all treatment groups were rated as mild to moderate (7 of 235 vortioxetine TEAEs [3.0%] were judged severe by the investigators). The rate of discontinuations because of TEAEs was relatively low in the vortioxetine group (3.6%) and comparable to that in the placebo group (3.7%). The TEAEs with an incidence of 5% or more in the vortioxetine group were nausea (vortioxetine, 20.4% placebo, 4.2% and duloxetine, 20.8%), headache (10.2%, 8.4%, and 11.6%, respectively), and diarrhea (5.6%, 2.6%, and 2.9%, respectively) (Table 3).

The incidence of serious TEAEs was low during the 8-week treatment period (*n*=4, five total events, two possibly attributed to treatment). One subject in the placebo group was hospitalized for worsening of depression that was possibly attributed to treatment. One subject in the vortioxetine treatment arm attempted suicide that was possibly attributed to treatment. No clinically significant findings within or differences between groups emerged regarding physical examination, hematology, clinical chemistry, ECG, vital signs, or C-SSRS. No deaths occurred in the study.

## Discussion

Many patients suffering from MDD present some form of dysfunction in certain cognitive domains; this affects >60% of all depressed patients and significantly impacts treatment outcomes (Bora *et al*, 2013; Gualtieri and Morgan, 2008; Lee *et al*, 2012; Murrough *et al*, 2011; Rock *et al*, 2014; Trivedi and Greer, 2014). In a study of newly diagnosed depressed (*n*=30) and healthy subjects (*n*=30), 63.3% of the depressed patients had cognitive difficulties compared with 3.3% of healthy controls, with attention/concentration being the most commonly affected domain of cognitive function, followed by memory disturbance (Afridi *et al*, 2011). Patients with depression in one study performed approximately one-half of SD worse than healthy subjects on verbal learning and memory tests (Burt *et al*, 1995).

Traditionally, performance-based neuropsychological tests have been used to evaluate the effect of antidepressant treatment on cognitive dysfunction. The relationship between these objective measures of cognitive performance (ie, neuropsychological tests) and subjective reporting of cognitive complaints (ie, self-rated assessment of cognitive dysfunction) is not well understood, and discrepancies between these ways of evaluating cognition have been reported in the literature. The neuropsychological tests used in this study were selected because they assess aspects of cognition shown previously to be most impaired in subjects with depression, specifically, verbal learning, verbal memory, attention, executive function, and working memory. This study also included a number of patient-reported measures of cognitive function as well as the UPSA to evaluate overall functional capacity. The primary efficacy end point—the number of correct symbols on the DSST—is designed to measure a variety of cognitive functions, including executive functioning, processing speed, attention, spatial perception, and visual scanning, and has been found to be one of the most robust predictors of outcome in patients with severe mental illness (Dickinson *et al*, 2007). The issue of which specific domains of cognition mediate performance on the DSST and other symbol coding tests has been discussed extensively in the literature, with no clear consensus on which among several domains, such as processing speed, executive functions, visuomotor processing, and working memory, is primary (Baudouin *et al*, 2009; Clarke *et al*, 2012; Dickinson *et al*, 2007; Heaton *et al*, 2001; Nuechterlein *et al*, 2004; Piccinin and Rabbitt, 1999; Salthouse, 1992; Satz *et al*, 2011; Stern, 2002; Zihl *et al*, 2014) Although the Measurement and Treatment Research to Improve Cognition in Schizophrenia (MATRICS) project Neurocognition Committee concluded that symbol coding tasks are best represented in the processing speed domain for schizophrenia studies (Nuechterlein *et al*, 2004), this is not necessarily the case with MDD and may be specific to data collected in patients with schizophrenia (Heaton *et al*, 2001). More recent analyses suggest that in non-schizophrenia populations, symbol coding tasks such as the DSST are mediated more by executive functions than any other domain, such as processing speed or visuomotor tracking (Baudouin *et al*, 2009; Parkin and Java, 2000; Piccinin and Rabbitt, 1999; Rosano *et al*, 2008; Salthouse, 1992).

The results of this multicenter, double-blind, placebo-controlled phase 2 trial indicate that vortioxetine is an efficacious and well-tolerated treatment for MDD in subjects with self-reported cognitive dysfunction. Vortioxetine produced significant improvement compared with placebo on the primary end point for the study, cognitive function as measured by the DSST performance. The DSST, a cognitive performance measure that assesses a variety of cognitive functions including processing speed and executive functions, has demonstrated strong correlation with overall cognitive functioning and functional outcomes in patients with acute depression (Raskin *et al*, 2007), schizophrenia (Dickinson *et al*, 2007), and bipolar disorders (Robinson *et al*, 2006). The improvement in cognitive functioning with vortioxetine, as demonstrated by the increase in DSST performance, may reflect an improvement in the reduced executive functions and slowed cognitive processing that are characteristic of many patients with MDD. These objective, performance-based improvements in cognition in MDD patients treated with vortioxetine may present on clinical examination as improved concentration, memory, and an ability to think more clearly.

Vortioxetine also improved performance on one of several secondary cognitive end points, Trail Making B, which measures processing speed and executive functions, and the PDQ, which provides a subjective assessment of cognitive function. Similar to previous studies, vortioxetine also demonstrated significant improvement in depression symptoms and overall clinical status. Path analysis indicated the cognitive performance benefits associated with vortioxetine are attributable to direct effects on cognition rather than a nonspecific effect of improvement in mood. Interestingly, the UPSA—a performance-based additional measure of functional capacity—showed significant improvement for vortioxetine compared with placebo. Together, these data provide support for the overall clinical effect of vortioxetine on depression, cognitive function, and functional capacity in adults with MDD. The results on the WLQ should be interpreted with caution because this workplace questionnaire was only performed in the subset of working patients (41.4%). Although neither vortioxetine nor duloxetine demonstrated clinical efficacy on the WLQ sensitivity index, vortioxetine was significantly different from placebo in reducing the difficulty of time management subscale of the WLQ.

Duloxetine was included in this study as an active reference to confirm the assay sensitivity to depressive symptoms. Although duloxetine treatment did significantly improve depression symptoms (validating the study, as assessed by improvements on the MADRS and CGI-I), it did not significantly separate from placebo on the DSST, UPSA, or any of the secondary cognitive measures. Duloxetine showed superiority over placebo on the PDQ and CPFQ; however, path analysis suggests any effect on cognitive function was attributable to improvement in depressive symptoms rather than a direct effect on cognition. No definitive conclusions can be drawn as to the relative benefit on cognitive effects of vortioxetine compared with duloxetine from this single trial, because the study was not appropriately powered for this analysis. Any determination of the relative merits of either compound for treatment of cognitive symptoms of depression would require further study, either by combining all the available evidence from multiple studies or in studies specifically designed to answer this question.

However, the results of the current clinical study confirm the clinical benefits for vortioxetine in MDD patients who self-reported cognitive dysfunction and broaden the understanding of the results seen in previous clinical studies. In studies in patients with MDD or generalized anxiety disorder, in which primary end points were change in depressive or anxiety symptoms, respectively, *post hoc* analysis showed that vortioxetine improved cognition subitems in the MADRS Item 6 (concentration difficulties) and Hamilton Anxiety Scale (HAM-A) Item 5 (concentration and memory) compared with placebo (Keefe *et al*, 2013b). In addition, predefined exploratory cognitive tests for verbal learning (Rey Auditory Verbal Learning Test (RAVLT)) and executive function, working memory, processing speed, and visuospatial attention (DSST) were included in a study of vortioxetine in elderly patients with MDD. In that study, the tested dose of vortioxetine 5 mg/day improved cognitive dysfunction as measured by the RAVLT and DSST, whereas duloxetine (included as an active reference) improved performance on the RAVLT but not the DSST (Katona *et al*, 2012). Furthermore, clinical results from a recently published double-blind, placebo-controlled study demonstrated the positive impact of vortioxetine on cognitive function in depressed nonelderly adults, with path analysis showing that up to two-thirds of the impact on cognitive function was a direct effect, independent of the amelioration of depressive symptoms (McIntyre *et al*, 2014). The preclinical and clinical data on vortioxetine suggest that it has procognitive properties that are unique to its mode of action. This intriguing notion requires further research to assess whether these findings are confirmed.

The reported effects of vortioxetine on cognitive functioning in patients with MDD provide potential insights into issues regarding the pseudospecificity of cognitive function treatment response in this population. Because a proportion of the effect on cognitive performance is often attributed to mood and may be reflective of a pseudospecific response, it is most relevant to adjust for the effect on depressive symptoms to be able to compare and interpret the independent effect of treatment on cognitive functioning. Although this was a clinical study of patients with acute depression (as evidenced by a baseline MADRS of ~31.5 across treatment groups), path analysis provides statistical evidence that the benefits of vortioxetine on cognitive functioning are primarily a direct treatment effect and not due to an improvement in general depressive symptoms. Path analysis is a well-established statistical approach to decompose correlations into different pieces for interpretation of treatment effects and, being an extension of a multiple linear regression analysis, helps to address and mitigate concerns regarding the pseudospecificity of these results (Arnold *et al*, 2012; Ditlevsen *et al*, 2005; Marangell *et al*, 2011; Streiner, 2005). By adjusting for the effect on the MADRS total score—which adds considerable variation to the overall results—the contribution of the effect of treatment of depressive symptoms is disregarded. In the current study, the direct treatment effect on cognitive functioning was 75.7% for vortioxetine, suggesting limited contribution to the improvement in mood symptoms in the overall benefits on cognition. Although the statistical approach of addressing pseudospecificity through path analysis may be limited compared with restricting a trial to remitted patients, the effects of vortioxetine on cognitive functioning seen in the path analysis are consistent with results seen in previous trials. Katona *et al* (2012) showed that more than two-thirds of the effect of vortioxetine on cognitive performance, measured using the DSST (83%) and RAVLT (acquisition 71% delayed recall 72%), was a direct effect rather than an indirect effect through an improvement in general depressive symptoms. In the results of a recently published placebo-controlled trial by McIntyre *et al* (2014), it was highlighted that the proportion of direct effect on the DSST was 66% and 56% for the vortioxetine 10 and 20 mg arms, with similar results on a composite *z*-score of change in DSST and change in RAVLT (64% and 48%, respectively).

The magnitude of the observed effect on cognitive dysfunction in MDD can be contextualized using standardized effect sizes. This allows for a comparison of the magnitude of the effect sizes between studies, the different cognitive tests, and the different versions of a given test. In assessing the magnitude of the effect on the neuropsychological tests, it is important to distinguish between the level of deficits within a disorder, the effect size following treatment (pretreatment *vs* posttreatment), and between-group effect size (drug–placebo). In line with this, the standardized effect sizes for vortioxetine should be interpreted in the context of cognitive dysfunction in MDD and in the context of between-group comparisons. The magnitude of cognitive impairment in patients with MDD is ∼0.2–0.7 SD below that of healthy patients (Rock *et al*, 2014; Tuulio-Henriksson *et al*, 2011). From the perspective of overall functional performance, the degree of cognitive dysfunction in patients with MDD is comparable to that after 24 h of sleep deprivation or with blood alcohol levels sufficient to be considered legally impaired (driving, and so on) (Goel *et al*, 2009). In this study and two previous studies in patients with MDD, vortioxetine significantly improved cognitive performance (as measured by the change in DSST–number of correct symbols) (Katona *et al*, 2012; McIntyre *et al*, 2014), with a Cohen's *d* effect size ranging from 0.25 to 0.48 across all three studies. Although the authors acknowledge that the overall effect size is relatively small for the treatment of patients with MDD, clinical meaningfulness of an effect cannot be directly determined by the magnitude of change (Keefe *et al*, 2013a; McGough and Faraone, 2009). The overall results of vortioxetine on cognitive function in MDD patients subjectively reporting cognitive dysfunction could be better viewed in context with the treatment of cognitive functioning in patients with Alzheimer's disease, where the 1-year treatment effect of cholinesterase inhibitors results in a Cohen's *d* effect size ranging from 0.3 to 0.5 (Atri *et al*, 2008; Rockwood, 2004), despite a magnitude of disease impact several SD above the norm.

The magnitude of cognitive dysfunction seen in MDD can significantly impair day-to-day functioning and have detrimental consequences for the patients in maintaining their expected psychosocial and work functioning. In contrast to individuals suffering from dementia or schizophrenia (Alzheimer's Association, 2012; Tan, 2009), individuals treated for MDD often return to a work environment, where the MDD-associated residual cognitive impairment can adversely impact performance (Adler *et al*, 2006). Because vortioxetine significantly improved performance on the DSST (number of correct symbols) in all three studies in patients with cognitive dysfunction, with a standardized effect size ranging from 0.25 to 0.48 across the studies, and because these effect sizes approach the magnitude of the cognitive deficit in MDD in general, it is likely that this amount of improvement is clinically relevant.

Finally, the current clinical study has limitations. The lack of formalized diagnostic inclusion criteria for cognitive dysfunction in patients with MDD may have led to the inclusion of patients without clinically relevant cognitive deficits. Although cognitive dysfunction is a part of the diagnostic criteria for MDD, and thus a core part of the illness, it is usually based upon clinician observation and patient self-report. In line with current practice and in order to evaluate the potential cognitive benefits of vortioxetine in a more representative population of MDD patients, this study included subjects who self-reported cognitive dysfunction but did not specifically require objectively assessed cognitive dysfunction for inclusion. Although this clinical study is limited in its reliance on self-reported cognitive dysfunction as opposed to a clear diagnostic basis for study inclusion, the screening process used to assess cognitive dysfunction is very similar to that which occurs in clinical practice, thus replicating experiences in real-world practice in a double-blind manner. Analysis of the baseline DSST performance scores suggests that the patient population suffered from clinically relevant objective symptoms of cognitive dysfunction. Additional evaluations of MDD patients with objectively assessed cognitive dysfunction may further enhance our understanding of the role that vortioxetine may play in treating MDD with cognitive dysfunction.

A further limitation is the lack of additional clinician-rated measures of patient functioning beyond the UPSA, a performance-based measure of patient functioning that correlates highly with cognitive outcomes. The results on the WLQ may not be generalizable, as the WLQ was only performed in the subset of patients who had been employed at least 14 days before baseline (219 of 529 subjects, 41.4%). Although neither vortioxetine nor duloxetine demonstrated clinical efficacy on the WLQ sensitivity index, vortioxetine was significantly different from placebo in reducing the difficulty of time management on the WLQ (–20.90 *vs* −12.78, respectively; *P*=0.045), a subscale closely correlated to cognitive functioning.

Finally, the current study was only powered to detect a statistical difference between vortioxetine and placebo on the primary efficacy outcome of change from baseline in DSST performance, limiting the ability to detect a statistically significant difference between vortioxetine and duloxetine on any other primary or secondary outcome measure. Although there was no statistical difference between vortioxetine and duloxetine on the DSST performance score, vortioxetine demonstrated statistical superiority to duloxetine on the UPSA composite score.

## Conclusions

In this study of adults with MDD and self-reported cognitive dysfunction, vortioxetine was statistically significantly superior to placebo on the predefined primary analysis, an objective measure of cognitive functioning, and on both predefined key secondary end points of global clinical status and patient-reported cognitive functioning. Vortioxetine was also significantly superior to placebo in the treatment of depressive symptoms and in the improvement of functional capacity. Duloxetine was not significantly different from placebo on objective measures of cognitive functioning or functional capacity but was significantly superior to placebo on depressive symptoms, global clinical status, and patient-reported cognitive functioning. Path analysis suggests that vortioxetine's beneficial impact on cognitive functioning in patients with MDD may be a direct treatment effect. Safety findings in this study were similar to those observed in previous trials of vortioxetine and duloxetine.

## Funding and Disclosure

Dr Mahableshwarkar is an employee of Takeda Pharmaceuticals and holds stock in GlaxoSmithKline, Johnson & Johnson, and Pfizer. Dr Zajecka has received grant/research support from Alkermes, Allergan, AstraZeneca, Cyberonics, ElMindA, Forest, Cheryl T Herman Foundation, Hoffman-LaRoche, Naurex, National Institutes of Health, Shire, and Takeda. He is currently a consultant to, or serves on advisory boards for, Avanir, Eli Lilly, Forest, H Lundbeck, PamLab, Shire, and Takeda. Dr Zajecka has also served as an expert witness for psychiatric testimony. Dr Jacobson is an employee of Takeda Pharmaceuticals and holds stock in Pfizer and United HealthCare. Dr Chen is an employee of Takeda Pharmaceuticals. Dr Keefe has currently or in the past 3 years received investigator-initiated research funding support from the Brain Plasticity, Department of Veteran's Affairs, Feinstein Institute for Medical Research, GlaxoSmithKline, National Institute of Mental Health, Novartis, PsychoGenics, Research Foundation for Mental Hygiene, and the Singapore National Medical Research Council. He has currently or in the past 3 years received honoraria from or has served as a consultant or advisory board member for Abbvie, Akebia, Amgen, Astellas, Asubio, AviNeuro/ChemRar, BiolineRx, Biogen Idec, Biomarin, Boehringer-Ingelheim, Eli Lilly, EnVivo, GW Pharmaceuticals, H Lundbeck, Helicon, Merck, Minerva Neurosciences, Mitsubishi, Novartis, Otsuka, Pfizer, Roche, Shire, Sunovion, Takeda, and Targacept. He receives royalties from the BAC testing batteries and the MATRICS Battery (BACS Symbol Coding). He is also a shareholder in NeuroCog Trials and Sengenix.

## Figures and Tables

**Table 1:** Baseline Demographics and Clinical Characteristics of Study Participants

	**Placebo (*n*=194)**	**Vortioxetine (*n*=198)**	**Duloxetine (*n*=210)**
*Age (years)*			
Mean (SD)	45.0 (12.1)	44.2 (12.2)	45.7 (11.5)
Range	18–64	18–65	20–65
			
*Gender,* n *(%)*			
Male	75 (38.7%)	63 (31.8%)	72 (34.3%)
Female	119 (61.3%)	135 (68.2%)	138 (65.7%)
			
*Race,* n *(%)*			
Caucasian	171 (88.1%)	169 (85.4%)	176 (83.8%)
Black	20 (10.3%)	28 (14.1%)	27 (12.9%)
Asian	1 (0.5%)	1 (0.5%)	6 (2.9%)
Other	2 (1.0%)	0	1 (0.5%)
			
*DSST, mean (SD)*			
Number of correct symbols	43.5 (12.1)	42.3 (11.7)	43.4 (12.1)
			
*CPFQ, mean (SD)*			
Total score	30.2 (4.5)	29.5 (5.3)	29.3 (4.9)
			
*PDQ, mean (SD)*			
Total score	43.9 (10.6)	43.5 (10.9)	41.2 (12.6)
			
*MADRS, mean (SD)*			
Total score	31.9 (3.8)	31.4 (3.9)	31.7 (3.8)
			
*CGI-S, mean (SD)*			
Score	4.6 (0.6)	4.6 (0.6)	4.6 (0.6)

**Table 2:** Efficacy Results at Week 8 (LS mean±SE (95% CI)) (ANCOVA, OC)

	**Placebo (*n*=167)**	**Vortioxetine 10–20 mg (*n*=175)**			**Duloxetine 60 mg (*n*=187)**		
	**Change from baseline**	**Change from baseline**	**Difference from placebo**	***P-*value**	**Change from baseline**	**Difference from placebo**	***P-*value**
*Primary end point*							
DSST–number of correct symbols	2.85±0.54	4.60±0.53	1.75±0.74 (0.28;3.21) (standardized effect size 0.254)	0.019	4.06±0.51	1.21±0.73 (−0.23;2.56) (standardized effect size 0.176)	0.099
							
*Predefined secondary end points*							
PDQ–attention/concentration and planning/organisation*	−6.3±0.57	−8.9±0.55	−2.6±0.78 (−4.1;−1.0)	0.001	−9.3±0.53	−3.0±0.77 (−4.5;−1.5)	<0.001
CGI-I score^*,**^	2.64±0.09	2.35±0.09	−0.29±0.12 (−0.53;−0.05)	0.017	2.24±0.08	−0.40±0.12 (−0.64;−0.17)	<0.001
							
*Secondary end points assessing cognitive dysfunction*							
Trail Making Test A (total time, s)	−6.65	−7.70	−1.05	0.446	−8.06	−1.41	0.303
Trail Making Test B (total time, s)	−9.06	−18.73	−9.67	<0.001	−14.60	−5.54	0.053
Stroop Congruent Test (time to completion, s)	−4.37	−3.30	1.07	0.482	−4.54	−0.18	0.904
Stroop In congruent test (time to completion, s)	−8.11	−8.17	−0.05	0.980	−9.83	−1.72	0.422
Groton Maze Learning Test (total errors)^†^	−3.49	−5.43	−1.94	0.311	−5.16	−1.67	0.378
Detection Task (Speed of Performance, Log10 msec)	−0.03	−0.05	−0.02	0.134	−0.04	−0.01	0.605
Identification Task (Speed of Performance, Log10 msec)	−0.02	−0.04	−0.02	0.102	−0.03	−0.01	0.426
One-Back Task (Speed of Performance, Log10 msec)	−0.02	−0.03	−0.01	0.467	−0.02	0	0.733
*Additional endpoints*							
MADRS Total Score*	−12.5±0.7	−14.8±0.7	−2.3±1.0 (−4.3;−0.4)	0.02	−15.8±0.7	−3.3±1.0 (−5.2;−1.4)	<0.001
UPSA composite score	5.07±0.59	8.01±0.57	2.94±0.81 (1.35;4.52)	<0.001	5.45±0.55	0.38±0.80 (−1.19;1.94)	0.637
UPSA–VIM composite score***	2.84±0.71	4.60±0.70	1.75±0.99 (−0.20;3.71)	0.078	4.01±0.64	1.17±0.95 (−0.70;3.04)	0.219
UPSA–Brief composite score***	6.99±0.89	11.00±0.87	4.02±1.21 (1.63;6.41)	0.001	6.64±0.88	−0.35±1.23 (−2.76;2.06)	0.775
CPFQ total score*	−6.9±0.51	−8.1±0.50	−1.2±0.70 (−2.6;0.2)	0.086	−8.7±0.48	−1.7±0.69 (−3.1;−0.4)	0.012
WLQ–Percentage Productivity Loss^†^	3.07±0.65	−4.41±0.64	−1.35±0.88 (−3.08;0.39)	0.127	−3.80±0.64	−0.73±0.86 (−2.44;0.97)	0.398
WLQ–Time Management^†^	−3.07±0.65	−20.90±2.89	−8.13±4.02 (−16.06;−0.20)	0.045	−15.30±2.99	−2.53±4.00 (−10.42;5.36)	0.528
WLQ–Physical Demand^†^	−4.23±4.09	−3.88±3.94	0.36±5.49 (−10.48;11.19)	0.948	−3.91±4.00	0.32±5.41 (−10.36;11.00)	0.953

Abbreviations: ANCOVA, analysis of covariance; OC, observed cases.

*MMRM, FAS.

**CGI–S baseline score utilized.

***UPSA–VIM assessed in US patients and UPSA–Brief assessed in EU patients.

^†^WLQ scores based on a sub–group of working patients (~40% of total study population).

**Table 3:** Incidence of Adverse Events (Frequency in ≥5.0% of Participants) in Subjects Treated with Vortioxetine, Duloxetine, or Placebo for 8 Weeks

	**Placebo, *n*=191**	**Vortioxetine, *n*=196**	**Duloxetine, *n*=207**
*Treatment-emergent adverse events (TEAEs), subjects,* n *(%)*			
Any TEAE	85 (44.5%)	117 (59.7%)	119 (57.5%)
TEAEs leading to discontinuation	7 (3.7%)	7 (3.6%)	13 (6.3%)
Serious TEAEs	2 (1.0%)	1 (0.5%)	1 (0.5%)
Serious TEAEs leading to discontinuation	1 (0.5%)	0	1 (0.5%)
			
*TEAEs with incidence of ≥5% in any treatment arm, subjects,* n (*%)*			
Nausea	8 (4.2%)	40 (20.4%)	43 (20.8%)
Headache	16 (8.4%)	20 (10.2%)	24 (11.6%)
Diarrhea	5 (2.6%)	11 (5.6%)	6 (2.9%)
Nasopharyngitis	11 (5.8%)	7 (3.6%)	8 (3.9%)
Dizziness	5 (2.6%)	6 (3.1%)	11 (5.3%)
Dry mouth	9 (4.7%)	6 (3.1%)	16 (7.7%)
Decreased appetite	1 (0.5%)	3 (1.5%)	12 (5.8%)
