# Supplementary figures and images for: A Randomized, Placebo-Controlled, Active-Reference, Double-Blind, Flexible-Dose Study of the Efficacy of Vortioxetine on Cognitive Function in Major Depressive Disorder
Source: Neuropsychopharmacology. 2015 Apr 1;40(8):2025–37. doi: 10.1038/npp.2015.52 (PMC4839526; doi:10.1038/npp.2015.52)

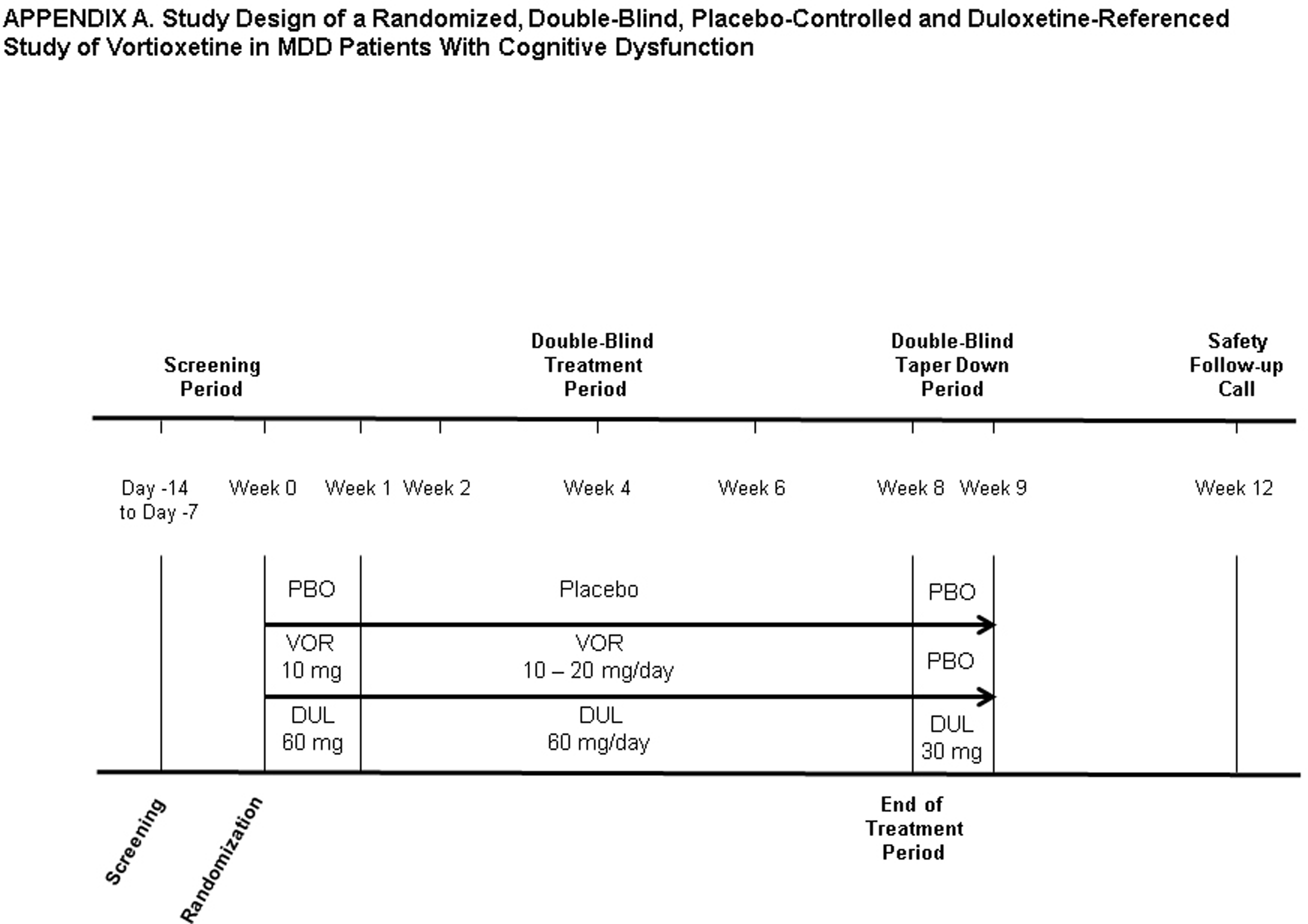

Supplement: Supplementary Appendix A [file npp201552x1.tif]

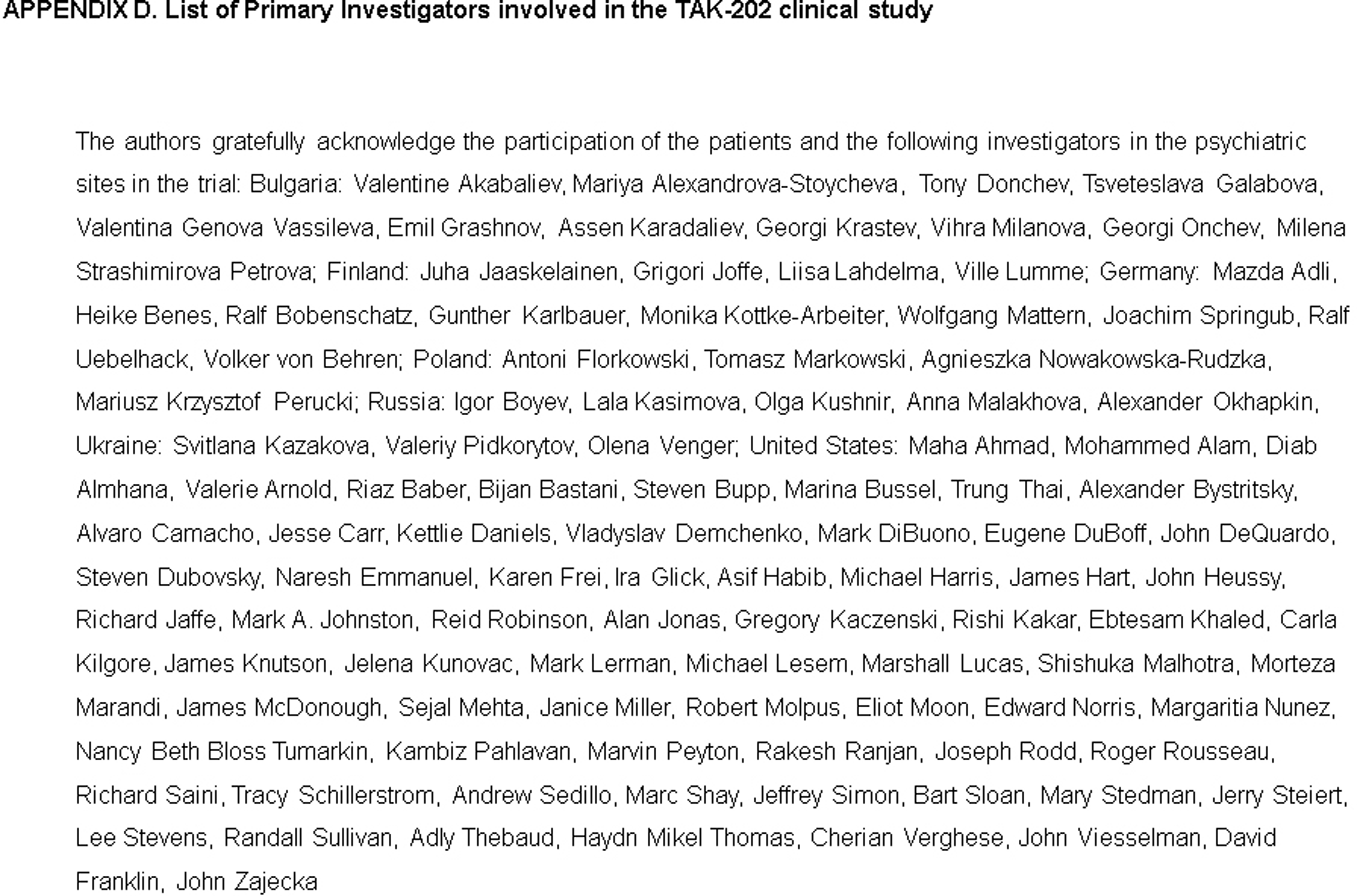

Supplement: Supplementary Appendix D [file npp201552x4.tif]
