# Supplementary material for: A Randomized, Placebo-Controlled, Active-Reference, Double-Blind, Flexible-Dose Study of the Efficacy of Vortioxetine on Cognitive Function in Major Depressive Disorder
Source: Neuropsychopharmacology. 2015 Apr 1;40(8):2025–37. doi: 10.1038/npp.2015.52 (PMC4839526; doi:10.1038/npp.2015.52)
Supplement: Supplementary Appendix B [file npp201552x2.doc]

APPENDIX B. Testing Hierarchy of Primary, Secondary, and Additional Endpoints of a Randomized, Double-Blind, Placebo-Controlled and Duloxetine-Referenced Study of Vortioxetine in MDD Patients with Cognitive Dysfunction

**Primary Endpoint**

• Change from baseline to week 8 in the DSST – Number of Correct Symbols (integrated cognitive functioning)

**Predefined Key Secondary Endpoints**

• Change from baseline to Week 8 in the PDQ attention/concentration and planning/organization subscore

• CGI-I score at Week 8

**Secondary Endpoints**

*Cognitive Symptoms*

• Change from baseline to week 8 in the Trail Making Test A (TMT A) (speed of processing)

• Change from baseline to week 8 in the Trail Making Test B (TMT B) (executive functioning)

• Change from baseline to week 8 in the Stroop Test (executive functioning)

• Change from baseline to week 8 in the Groton Maze Learning Test (executive function, visual learning and memory)

• Change from baseline to week 8 in the Detection Task (motor speed)

• Change from baseline to week 8 in the Identification Task (attention)

• Change from baseline to week 8 in the One-Back Task (attention, working memory)

• Change from Baseline to Week 8 in the DSST score in MADRS nonresponders and nonremitters

• Path analysis estimate of the proportion of cognitive dysfunction improvement from baseline to week 8 attributable to direct effects and indirect effects via depressive symptom change using the MADRS total score and the DSST performance score

*Depressive symptoms*

• Change from baseline to week 8 in the MADRS total score and individual item scores

• Response rates (defined as a >50% decrease in MADRS total score from baseline) at week 8

• Remission rates (defined as a MADRS total score <10) at week 8

*Global Clinical Status*

• Change from baseline to week 8 in the CGI-S score

**Additional Endpoints**

• Change from baseline to all visits in the following:

o MADRS total score

o MADRS response and remission rates

o CGI-S score

o CGI-I score

• Change from baseline to week 8 in the Perceived Deficits Questionnaire (PDQ) total score and attention/concentration, retrospective memory, prospective memory, and planning/organization subscores (patient-reported cognitive function)

• Change from baseline to week 8 in the Cognitive and Physical Functioning Questionnaire (CPFQ) total score and physical dimension and cognitive dimension subscores (patient- reported cognitive functioning)

• Change from baseline to week 8 in the University of San Diego Performance-Based Skills Assessment (UPSA) (performance measures of functional capacity)

• Change from baseline to week 8 in the percentage of productivity loss as measured by the Working Limitation

Questionnaire (WLQ) (patient-report workplace productivity)
