# Supplementary material for: A Randomized, Placebo-Controlled, Active-Reference, Double-Blind, Flexible-Dose Study of the Efficacy of Vortioxetine on Cognitive Function in Major Depressive Disorder
Source: Neuropsychopharmacology. 2015 Apr 1;40(8):2025–37. doi: 10.1038/npp.2015.52 (PMC4839526; doi:10.1038/npp.2015.52)
Supplement: Supplementary Appendix C [file npp201552x3.ppt]

## Slide 1
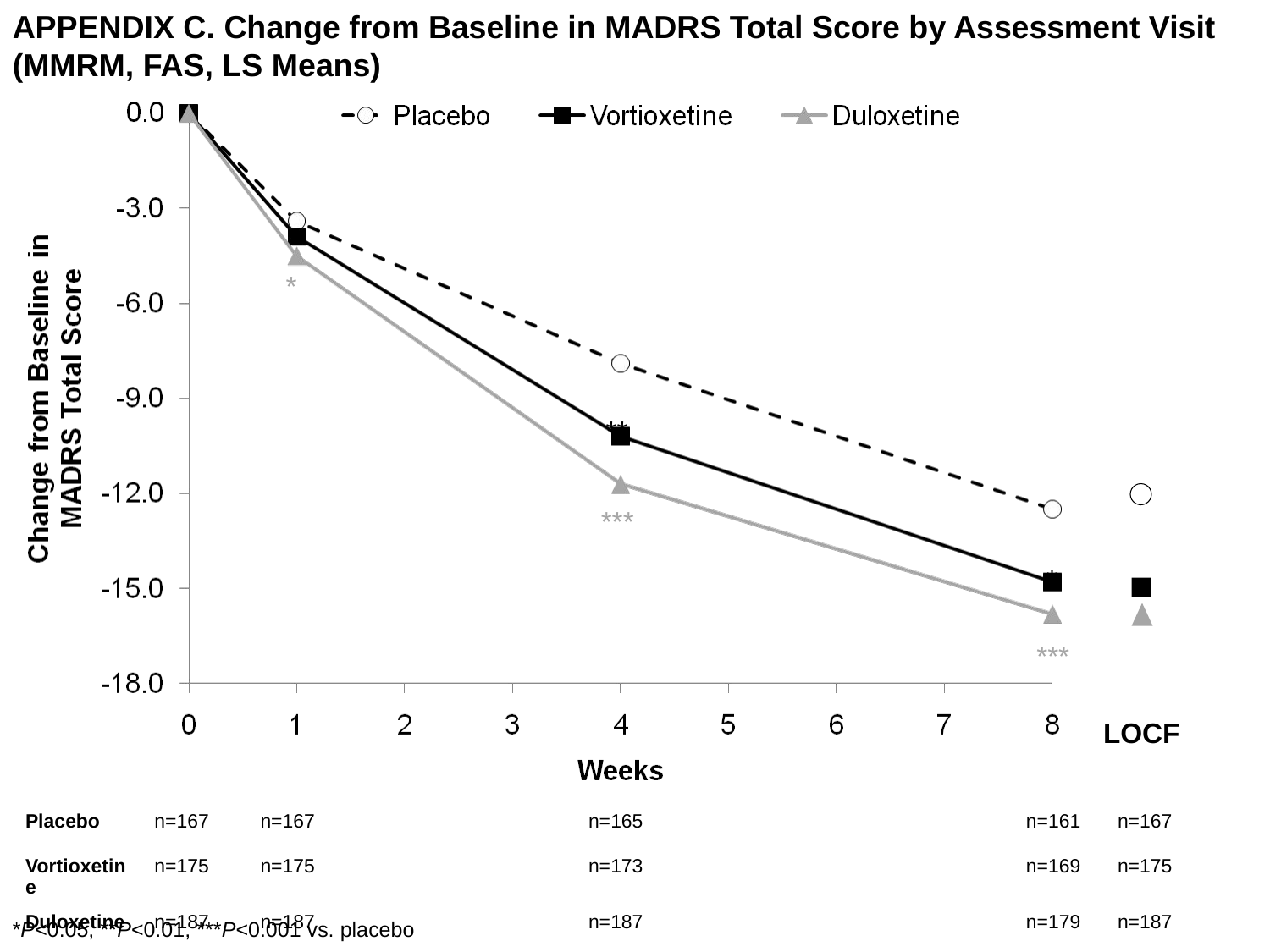

# APPENDIX C. Change from Baseline in MADRS Total Score by Assessment Visit (MMRM, FAS, LS Means)
*
**
***
*
***
LOCF
*P<0.05, **P<0.01, ***P<0.001 vs. placebo
| Placebo | n=167 | n=167 | | | n=165 | | | | | n=161 | n=167 |
| --- | --- | --- | --- | --- | --- | --- | --- | --- | --- | --- | --- |
| Vortioxetine | n=175 | n=175 | | | n=173 | | | | | n=169 | n=175 |
| Duloxetine | n=187 | n=187 | | | n=187 | | | | | n=179 | n=187 |
