# Supplementary material for: A Randomized, Placebo-Controlled, Active-Reference, Double-Blind, Flexible-Dose Study of the Efficacy of Vortioxetine on Cognitive Function in Major Depressive Disorder
Source: Neuropsychopharmacology. 2015 Apr 1;40(8):2025–37. doi: 10.1038/npp.2015.52 (PMC4839526; doi:10.1038/npp.2015.52)
Supplement: Supplementary Information [file npp201552x5.doc]

**Appendices**

APPENDIX A. Study Design of a Randomized, Double-Blind, Placebo-Controlled and Duloxetine-Referenced Study of Vortioxetine in MDD Patients With Cognitive Dysfunction

APPENDIX B. Testing Hierarchy of Primary, Secondary, and Additional Endpoints of a Randomized, Double-Blind, Placebo-Controlled and Duloxetine-Referenced Study of Vortioxetine in MDD Patients With Cognitive Dysfunction

APPENDIX C. Change From Baseline in MADRS Total Score by Assessment Visit (MMRM, LS Means)

APPENDIX D. List of Primary Investigators Involved in the TAK-202 Clinical Study
